# Formulation Optimization of Gluten-Free Functional Spaghetti Based on Maize Flour and Oat Bran Enriched in β-Glucans

**DOI:** 10.3390/ma4122119

**Published:** 2011-12-08

**Authors:** Lucia Padalino, Marcella Mastromatteo, Grazia Sepielli, Matteo Alessandro Del Nobile

**Affiliations:** 1 Istituto per la Ricerca e le Applicazioni Biotecnologiche per la Sicurezza e la Valorizzazione dei Prodotti Tipici e di Qualità, University of Foggia, via Napoli, Foggia 25–71100, Italy; E-Mails: l.padalino@unifg.it (L.P.); ma.mastromatteo@unifg.it (M.M.); 2 Department of Food Science, University of Foggia, via Napoli, Foggia 25–71100, Italy; E-Mail: g.sepielli@unifg.it

**Keywords:** maize, β-glucans, hydrocolloids, rheological characteristics, sensorial properties

## Abstract

The aim of this work concerns the manufacturing process of gluten-free functional spaghetti based on maize flour and oat bran, enriched with β-glucans (22%). More specifically, the goal of the study was to obtain oat bran-loaded maize spaghetti with sensory properties close to unloaded pasta. To this aim, the study has been organized in two subsequent trials. In the first one, the oat bran amount added to spaghetti was continuously increased until the overall sensory quality of pasta reached the set sensory threshold (oat bran concentration = 20%). The second experimental step was aimed to improve the overall sensory quality of oat bran loaded maize spaghetti. In particular, an attempt was made to increase the sensory quality of spaghetti added with 20% oat bran by means of structuring agents. To this aim, the effects of different kinds of some hydrocolloids and egg white powder on the rheological properties of dough, as well as on quality attributes of pasta were examined. The rheological analysis showed that the addition of hydrocolloids and white egg to the dough enriched with 20% oat bran did not cause any substantial difference in the viscoelastic properties, compared to samples without any structuring agents. The best overall quality for both fresh and dry spaghetti was obtained by the addition of carboxymethylcellulose and chitosan at a concentration of 2%.

## 1. Introduction

Pasta is a stable food product that is produced mainly by mixing durum wheat semolina and water. It can be consumed after cooking as fresh pasta or can also be dried for future use. Common pasta produced with wheat has better quality parameters (low cooking loss, firm pasta structure, decreased adhesiveness, *etc*.). In pasta processing, gluten is mainly responsible for the formation of the structure. Gluten is considered to be the most significant factor related to pasta cooking quality [1,2]. Gluten consists of gliadin and glutenin and is responsible for elasticity and al dente chewability of pasta, which is highly appreciated by consumers. However, some people with a specific genetic nature suffer from celiac disease upon consumption of food containing wheat, rye or barley [3]. The cause of this disease is the ingestion of dietary gluten, which may affect absorption of important nutrients such as iron, folic acid, calcium, and fat-soluble vitamins. The patients have intolerance against the gliadin fraction of wheat and the prolamins of rye (secalins) and barley (hordeins) [3]. The only effective treatment is a life-long gluten free diet [4,5,6,7,8,9].

Diets that contain moderate quantities of cereal grains, fruits and vegetables are likely to provide sufficient fiber. Due to the fact that gluten-free products generally are not enriched/fortified, and are frequently made from refined flour or starch, they may not contain the same levels of nutrients as the gluten-containing counterparts they are intended to replace. In order to obtain good quality pasta from alternative materials it is often necessary to modify the traditional production process [10]. In particular, balanced formulations and adequate technological production processes have to be adopted to counteract any changes in the rheological properties caused by the incorporation of these new ingredients [11]. Therefore, the amount of non-conventional flours (quinoa, amaranth, oat, soybean, maize, *etc*.) that can be added to or substituted for semolina represents a compromise between nutritional improvement of the pasta and achievement of satisfactory sensory properties [11].

Maize is recommended as a safe food for celiac patients since it possesses no gluten and can be used in the production of pasta. When maize is the only material used in pasta production, it requires new and efficacious starch organization able to substitute for the gluten network in the final product [12,13]. The addition of an aliquot of pregelatinized flour or starch that promotes, during the drying cycle, the formation of a starch network capable of improving pasta quality is a way to modify or improve the rheological properties of a formulation [13]. In the literature, it has also been reported that a substance suitable to produce a cohesive structure can overcome the absence of gluten. In this regard, several additives/ ingredients, including modified starch, gums or hydrocolloids, protein, and enzymes were used [14].

Dairy proteins and hydrocolloids can be used to mimic the viscoelastic properties of gluten and result in improved structural feel in the mouth, acceptability and shelf life [15]. Hydrocolloids, commonly named gums, are able to modify overall quality of the food product. Huang *et al.* [16] produced gluten free pasta with characteristics most similar to wheat-based pasta containing higher levels of modified starch, xanthan gum and locust bean gum.

Egg whites are an interesting food ingredient due to their multifunctional properties. It is well known that egg proteins, especially albumen proteins, form a reticule which can guarantee a cohesive mass, with a good consistency, achieved by thermal denaturation, even in the absence of gluten [17].

The relationship between food and health has an increasing impact on food innovation due to the popularity of the concept of functional food. In recent years, dietary fiber has received increasing attention from researchers and industry due to the likely beneficial effects on the reduction of coronary heart-related diseases, diabetes incidence and gut neoplasia [18]. The enrichment of gluten-free products with dietary fibers has proved to be necessary, since it has been reported that celiac patients generally have a low intake of fibers attributed to their gluten-free diet [19], therefore, it has been a topic of research for various teams of technologists (Codex Alimentarius Commission, 2000). One of these ingredients used by the food industry, classed as a dietary fiber, is β-glucan, a non-starch polysaccharide that it is located in the endosperm cell walls of oat constituting about 75% of endosperm cell walls. It is also present in the aleurone cell wall lesser in endosperm [20]. Oat has recently attracted research and commercial attention mainly due to its high content of β-glucans and compounds with antioxidant activity [21,22,23,24]. The health effects of β-glucans as related to decreasing cholesterol, improving gastrointestinal function and glucose metabolism would be achieved with a daily consumption level of 10 g β-glucans [25]. Conventional oat-based products contain 35–57 g of β-glucans per kg of product [26], which means that the daily consumption of adequate amounts of β-glucans is difficult from such products. The production of oat-based foods is therefore desirable in order to help meet the daily requirements for β-glucans consumption. On the basis of these assumptions, oat flour represents a good initiation in the making of functional foods.

This work was focused on the formulation optimization of spaghetti based on maize flour and oat bran enriched in β-glucans (22%). To this aim, the study has been organized in two subsequent steps: in the first step the oat bran amount added to spaghetti was continuously increased until the overall sensory quality of oat bran loaded maize based spaghetti decreased to the sensory threshold value, which was reached at an oat bran concentration of 20%. The second experimental step was aimed to improve the overall sensory quality of oat bran loaded maize spaghetti by means of structuring agents. The effects of the different amount of oat bran and the different kinds of selected hydrocolloids on the dough rheological properties as well as on quality attributes of pasta were examined.

## 2. Materials and Methods

### 2.1. Spaghetti Preparation

The heat-treated maize flour was bought from Bongiovanni mill (Mondovì, Cuneo, Italy) while oat bran enriched with β-glucans (22%) was purchased at the company of Di Minno Dario & C.S.r.l. (Milano, Italy). To prepare non-conventional dough a portion of maize flour was pregelatinized according the following procedure. In a steam cooker (LT50 2E Namad, Rome, Italy), 10 L of water was mixed with 10% (w/w) of flour and heated to 80 °C for about 1 hour. Subsequently, in order to prepare non-conventional pasta the pregelatinized flour was cooled to 40 °C and then was added to the mixture of the remaining maize flour (90% w/w), 1% (w/w) monoglycerides and oat bran rich in β-glucans and kneaded for 20 min.

In the first experimental phase, the oat bran was added at various concentrations from 5% to 20%. Then, in the second experimental phase the formulation of maize flour and 20% of oat bran was added with 2% of hydrocolloids dissolved during the pregelatinization process. In particular, Xantana Gum (named as XAN), Carboxymethylcellulose (CMC), Hydroxypropylcellulose (HPC), Agar (named as AG), Egg Protein powder (named as ALB), Tapioca starch (named as TAP), Guar seed flour (named as GUAR), Chitosan (named as CHIT) were used (all the hydrocolloids were bought from Farmalabor s.r.l—Canosa di Puglia, Italy, with the exception of Egg Protein powder bought from Molino Bongiovanni, Villanova, Mondavi, Italy). In order to dissolve the Chitosan, a solution at 0.5% of lactic acid was prepared (Chimpex Industriale S.P.A.). Moreover, the formulation of maize flour and 20% of oat bran added with Egg Protein powder was prepared without pregelatinized starch.

The dough based on maize flour (100%) was also manufactured and used as a reference (CTRL). Furthermore, commercially available spaghetti made of flour of maize (100%) and monoglycerides (Molino di Ferro, Treviso, Italy) named CM was also used as a reference.

Spaghetti samples were produced to 25 °C by means of a pilot plant made of an extruder (60VR, Namad, Rome, Italy) for the production fresh-extruded spaghetti and a dryer (SG600, Namad) for the production of dry spaghetti. The extrusion pressure was about 3.4 bar, whereas the temperature of the spaghetti after the extrusion was about 27–28 °C. The extruder was equipped with a screw (30 cm in length, 5.5 cm in diameter) which ended with a bronze die (diameter hole of 1.70 mm). The screw speed was 50 rpm.

Regarding the drying, the process conditions applied were the following: 1 step, time 20 min at 55 °C; 2 step, time 580 min at 75 °C; 3 step, time 40 min at 60 °C; 4 step, time 20 min at 45 °C; 5 step, time 840 min at 40 °C.

### 2.2. Dough Rheological Properties

Elongation and shear viscosity of each dough sample were investigated by means of a Rosand capillary rheometer (Malvern Instruments, Malvern, Worcester, UK) equipped with twin cylinders.

The samples used for the evaluation of rheological properties ware the flour/additive mixture produced from extruder, taken after die and without shape.

Two different length dies with the same diameter (1 mm) were selected to measure the entry pressure losses. The length of left die was of 10 mm and the pressure was of 10 psi. Whereas, the length of right die was of 0.25 mm and the pressure was of 150 psi. The experiments were carried out at 30 °C and at shear rate between 10–2,000 s^−1^. The rheological behavior is studied using the following power law model that satisfactory fitted the experimental data:

(1) $$\tau_{S}=K\cdot {\dot{\gamma }}_{S}^{n}$$

and

(2) $$\tau_{e}=L\cdot {\dot{\gamma }}_{e}^{m}$$

Where τ_s_ and τ_e_ are the shear stress [Pa] and extensional stress [kPa], K and L are the consistency indices [Pa∙s^n^ and kPa∙s^n^, respectively], the ${\dot{\gamma }}_{S}$ and ${\dot{\gamma }}_{e}$ are the shear and extension rate [1/s], and n and m are the flow indices (dimensionless). The elongation and shear viscosity (η_e_ and η_s_, respectively) were calculated on the range of shear and extension rate tested by using the following power law model [27,28]:

(3) $$\eta_{S}=K\cdot {\dot{\gamma }}_{S}^{n-1}$$

and

(4) $$\eta_{e}=L\cdot {\dot{\gamma }}_{e}^{m-1}$$

The Bagley correction was applied to all data from Rosand rheometer. Three measurements of the viscosity experiment were performed on each sample of the two batches.

### 2.3. Sensory Analysis

Fresh-extruded and dry spaghetti samples were submitted to a panel of ten trained tasters in order to evaluate the sensorial attributes. The panelists were selected on the basis of their sensory skills (ability to accurately determine and communicate the sensory attributes, appearance, odor, flavor and texture of a product). The panelists were however trained in sensory vocabulary and identification of particular attributes, prior to testing, by evaluating commercial conventional and non-conventional pasta. The panelists were asked to indicate color, homogeneity, resistance to break and overall quality of non-cooked spaghetti. The color measured against the pleasant characteristic yellow of pasta. The homogeneity is a measure of the presence of spots of different colors from that characteristic of the dough. The resistance to break is a measure of the strength with which the spaghetti is opposed to breakage. Elasticity, firmness, bulkiness, adhesiveness, color, odor, taste and overall quality of cooked spaghetti were also evaluated. The elasticity is a measure of the degree of extension of the spaghetti before the break and is evaluated on a spaghetti single practicing a slight traction in two points distant 10 cm. The firmness, the resistance of cooked pasta to compression by the teeth, was measured by compressing the spaghetti strand against the palate with the tongue. The bulkiness is a measure of the degree of jamming among the spaghetti strands and it has been evaluated by placing two spaghetti strands together and determining the force required for detachment. The adhesiveness is related to the formation of a surface coating made of amylose and it was evaluated by placing the spaghetti in the mouth, pressing it against the palate and determining the force required to remove it with the tongue. The odor is related to the pleasant of the sensations perceived by olfaction whereas the taste is related to the pleasant to the sensations perceived during mastication. Moreover, each spaghetti sample was cooked at different times and tested by the panel to estimate the optimal cooking time that was 10 min for the dry samples and 3 min for the fresh samples.

To this aim, a nine-point hedonic rating scale, where 1 corresponded to extremely unpleasant, 9 to extremely pleasant and 5 to satisfactory was used to quantify each attribute [14,29].

### 2.4. Statistical Analysis

The rheological and sensorial properties of gluten–free spaghetti based on maize flour enriched with oat bran as affected by different amount of oat bran and different kinds of hydrocolloids were evaluated in this study. The results of the rheological and sensorial analysis were compared by a one-way variance analysis (ANOVA). A Duncan’s multiple range test, with the option of homogeneous groups (p < 0.05), was carried out to determine significant differences between spaghetti samples. STATISTICA 7.1 for Windows (StatSoft, Inc, Tulsa, OK, USA) was used for this aim.

## 3. Results and Discussion

In the following, each of the two experimental steps to manufacture of gluten-free functional spaghetti based on maize flour and oat bran enriched in β-glucans (22%) are presented separately. The rheological properties of the different typologies spaghetti as well as the sensory characteristics of dry and fresh samples was presented and analyzed.

### 3.1. Step 1—Spaghetti of Maize Enriched with Oat Bran

#### 3.1.1. Rheological Measurement

Rheology is of considerable importance in the manufacture of various foods as it influences the machinability, processing conditions and quality of products [30]. Capillary rheometers can be used to determine a range of material functions. They can quickly and easily measure the flow properties of a material over the full range of forces, pressures, geometry, and temperatures that are encountered in real processes. A capillary rheometer was used in this work to determine extensional and shear viscosity that is the measure of the resistance of a fluid that is being deformed, by either shear stress or extensional stress. In fact, during the extrusion process are necessary levels of extensional and shear viscosity sufficient to allow the additives to bind to compounds of the flour (protein and starch) and then to develop a structure able to improve the mechanical properties of the pasta.

In the first experimental step, a progressive increase in the concentration of oat bran rich in β-glucans (5%, 10%, 15%, 20%) was used for the manufacture of gluten-free pasta with a high nutritional value.

**Figure 1:**
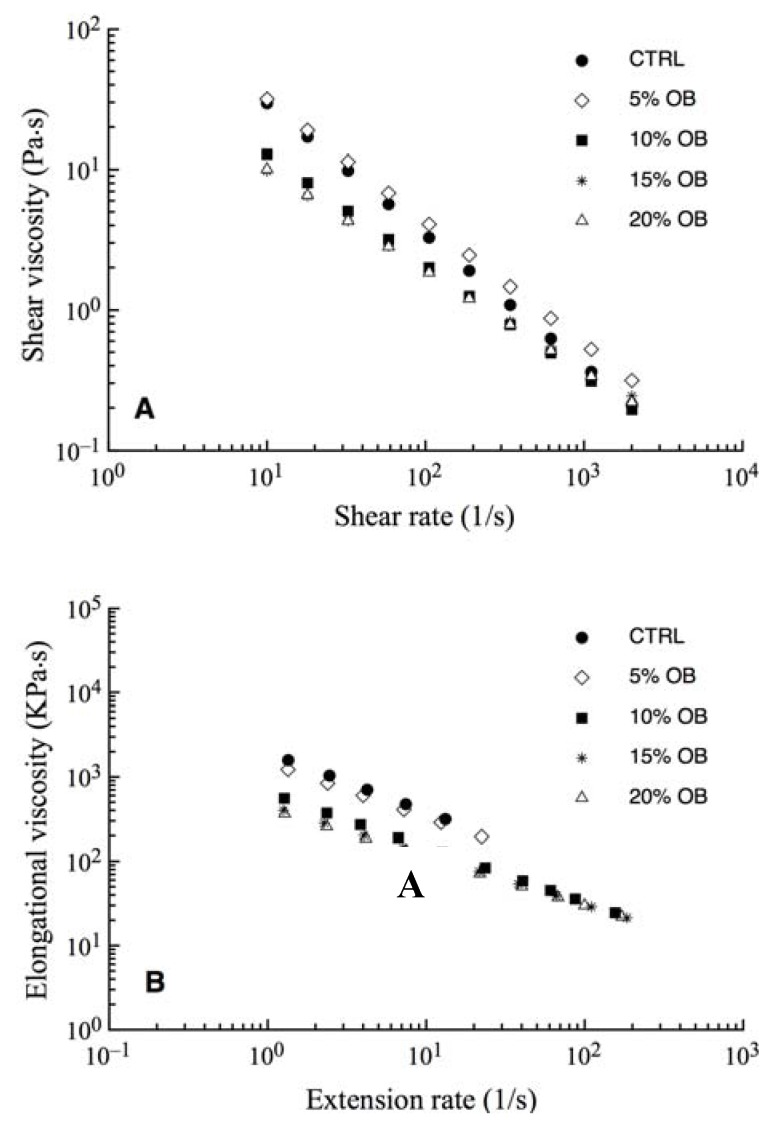
Rheological behavior of the dough samples with different concentration of oat bran. (**A**) Shear viscosity as a function of shear rate; (**B**) Elongation viscosity as a function of extension rate.

Figure 1 shows shear and elongation viscosity as a function of shear rate and extension rate, respectively. As can be inferred from the above figure, the trend is typical of pseudoplastic materials. In fact, shear and elongation viscosity values decline with the shear and extension rate. The pseudoplastic behavior of a polymeric system can be explained as follow [31]. If the system of asymmetric particles, which are initially randomly dispersed, is subjected to shear, the particles tend to align themselves with the major axis, in the direction of shear, thus reducing the viscosity. The degree of alignment is a function of the deformation rate. At low shear rate, there is only a slight departure from randomness but at higher shear rate particles are almost completely oriented [32]. From Figure 1, it can be seen that the CTRL and 5% OB samples had the higher values of elongation and shear viscosity with respect to the other samples. Coherently, the consistency indices K and L (Table 1), obtained by fitting Equation 1 and 2 to the experimental data, decreased with increasing percentage of oat bran in the pasta. In fact, the CTRL and 5% OB samples recorded the highest values of both the consistency indices K and L (Table 1), whereas for the samples 15% OB and 20% OB the lowest values were observed.

**Table 1:** Values of the consistency indices (L and K) and the flow indices (m and n) obtained by fitting Equations 1 and 2 to the experimental data.

	*L*	*m*	*K*	*n*
CTRL	1957.52^a^	0.2921^a^	253.87^a^	6.56E-02^a^
5% OB	1485.58^b^	0.3472^a^	235.43^a,b^	0.1291^a,b,c^
10% OB	649.46^c^	0.3513^a^	79.25^a,b^	2.09E-01^a,b^
15% OB	458.11^d^	0.4123^b^	48.49^b^	0.3033^c^
20% OB	420.90^d^	0.4260^b^	53.09^b^	0.278^b,c^

^a–d^ Mean in the same column followed by different superscript letters differ significantly (p < 0.05).

According to Manthey *et al.* [33], the decrease of dough viscosity influences both the mechanical energy required to extrude and the rate of dough extrusion. The authors suppose that the effect of reducing the viscosity of the pasta from the bran is due to the interruption of the continuity of the gluten matrix. In this case, most probably the oat bran interferes with the pregelatinized maize starch. Moreover, the dough viscosity is directly influenced by the amount of pregelatinized maize starch in the feed materials [34]. Therefore, the increase of the oat bran percentage and the consequent decreasing of the amount of maize flour used decreases the quantity of pregelatinized starch available to structure the dough and then decreases the firmness of dough.

#### 3.1.2. Sensory Analysis

The sensorial properties of the investigated samples were determined by means of a group of trained panelists and the results are listed in Table 2 and Table 3 for fresh-extruded and dry spaghetti respectively. The sensorial data on the pasta samples produced in the first experimental step showed that for the fresh extruded CTRL sample (non-cooked and cooked spaghetti) the overall quality was scored with the highest value with respect to the other spaghetti samples.

Table 2 shows that the overall quality decreased with the increase of the amount of oat bran [35]. In fact, the score of sensorial attributes such as firmness, elasticity, bulkiness and adhesiveness decreased with the increase of the oat bran amount; as a consequence the spaghetti overall quality was also found to be a decreasing function of bran oat concentration. In particular, the overall quality of spaghetti containing the highest amount of oat bran (20%) appeared to be much lower in comparison with CTRL sample, in fact, it was scored around the acceptability threshold (*i.e.*, five).

The sensory properties of the dry spaghetti samples are listed in Table 3. In particular, the sensorial attributes of cooked and non-cooked spaghetti were determined. Regarding the non-cooked spaghetti, all samples had positive score of overall quality. Moreover the CTRL is statistically similar to the CM reference sample. In particular, the CTRL sample showed the highest score because of the highest resistance to break value caused by the incorporation of an aliquot of pregelatinized starch. In fact, the addition of an aliquot of pregelatinized starch promotes the formation of a starch network capable of improving pasta cooking quality and the rheological properties of the dough sample [13]. This result is correlated to that obtained for the rheological properties; in fact the CTRL dough samples showed the higher values of the elongation and shear viscosity and then higher values of the consistency indices compared to the other doughs.

In fact, the increase in the oat bran content brings a decreasing elasticity and firmness of the spaghetti if compared to the CTRL sample. For example, the resistance to break for spaghetti with 20% oat bran decreased from 8.0 (CTRL) to 7.0. This result could be due to the fact that the presence of oat bran prevents the formation of the starch network negatively influences the pasta structure. On the other hand, the presence of oat bran did not negatively influence the value of odor and homogeneity of the investigated samples.

The sensorial data on the dry cooked spaghetti samples showed that the overall quality of the CTRL and CM samples have the highest values among the investigated spaghetti samples. For example, for samples with 15% and 20% oat bran the overall quality was found to be around the acceptability threshold with values of 5.6 and 5.4, respectively. In particular, the sample with 20% oat bran was at the limit of acceptability for the sensorial attributes such as firmness, elasticity and bulkiness; whereas, the adhesiveness was found to be still acceptable. This result could be due to the fact that the increase of the oat bran percentage and consequent decreasing of the amount of maize flour used decreased the quantity of pregelatinized maize starch available to structure the dough. This result is correlated to that obtained for the rheological properties; in fact the sample with 20% oat bran showed lower value of the consistency index compared to the other doughs and then a decrease of the firmness of dough. On the contrary, odor, taste and homogeneity of the sample with 20% oat bran were more than acceptable.

In conclusion, the results obtained in the first experimental step highlighted that the spaghetti enriched with the highest oat bran concentration had a score below or close to acceptability threshold for most of the examined sensorial attributes. Therefore, in order to improve the sensorial quality of maize pasta with 20% oat bran, hydrocolloids and white egg were used in the next experimental step.

**Table 2:** Sensory characteristics of fresh-extruded, cooked and non-cooked, spaghetti samples at different concentration of oat bran.

**Sample**	**Non-cooked spaghetti**				**Cooked spaghetti**									
	Color	Homogeneity	Odor	Overall Quality	Elasticity	Firmness	Fibrous	Bulkiness	Adhesiveness	Color	Homogeneity	Odor	Taste	Overall Quality
**CTRL Extruded**	7.5^a^ ± 0.17	8.8^a^ ± 0.27	8.1^a^ ± 0.24	8.0^a^ ± 0.17	6.6^a^ ± 0.24	6.7^a,b^ ± 0.26	8.0^a^ ± 0.17	6.7^a^ ± 0.26	6.2^a^ ± 0.26	7.0^a,b^ ± 0.17	8.0^a^ ± 0.17	7.2^a,b^ ± 0.26	7.0^a^ ± 0.17	7.1^a,b^ ± 0.24
**5% OB Extruded**	7.3^a,b^ ± 0.22	7.8^b^ ± 0.27	7.3^b,c^ ± 0.25	7.2^d^ ± 0.27	6.6^a^ ± 0.24	7.0^b^ ± 0.18	8.1^a^ ± 0.38	7.1^a,b^ ± 0.24	6.3^a^ ± 0.27	7.4^c^ ± 0.18	7.8^a^ ± 0.27	7.8^b^ ± 0.27	7.0^a^ ± 0.18	7.3^b^ ± 0.27
**10% OB Extruded**	6.7^c^ ± 0.26	6.8^c^ ± 0.27	7.0^c^ ± 0.18	7.0^d^ ± 0.17	5.7^e,d^ ± 0.27	6.0^d^ ± 0.18	7.0^c^ ± 0.18	7.8^c^ ± 0.24	6.6^b,c^ ± 0.24	7.0^a,b^ ± 0.18	6.7^b^ ± 0.26	7.0^c^ ± 0.18	7.0^a^ ± 0.18	6.2^e,f^ ± 0.27
**15% OB Extruded**	7.1^a,b,c^ ± 0.23	6.5^c^ ± 0.38	7.5^a,b^ ± 0.27	7.0^d^ ± 0.18	5.7^d,e^ ± 0.26	5.7^d,e^ ± 0.27	5.2^d^ ± 0.38	7.0^a,b^ ± 0.18	6.0^a^ ± 0.18	6.6^b^ ± 0.23	7.0^b^ ± 0.18	7.5^a,b^ ± 0.18	6.8^a^ ± 0.23	5.4^c^ ± 0.18
**20% OB Extruded**	7.0^b,c^ ± 0.23	6.4^c^ ± 0.23	7.0^c^ ± 0.38	6.4^c^ ± 0.18	5.4^e^ ± 0.23	5.4^e^ ± 0.23	4.4^b^ ± 0.23	7.0^a,b^ ± 0.18	6.1^a^ ± 0.23	6.5^b^ ± 0.38	7.0^b^ ± 0.18	6.8^c^ ± 0.23	6.9^a^ ± 0.23	5.0^g^ ± 0.18

^a–g^ Mean in the same column followed by different superscript letters differ significantly (p < 0.05).

**Table 3:** Sensory characteristics of dry cooked and non-cooked spaghetti samples at different concentration of oat bran.

**Sample**	**Non-cooked spaghetti**				**Cooked spaghetti**									
	Color	Homogeneity	Resistance to break	Overall Quality	Elasticity	Firmness	Fibrous	Bulkiness	Adhesiveness	Color	Homogeneity	Odor	Taste	Overall Quality
**CTRL**	8.0^a^ ± 0.18	7.4^a^ ± 0.18	8.0^a^ ± 0.18	8.0^a^ ± 0.18	7.3^a^ ± 0.38	7.7^a^ ± 0.40	8.0^a^ ± 0.18	7.5^a^ ± 0.40	8.0^a^ ± 0.18	7.5^a^ ± 0.40	8.0^a^ ± 0.18	7.7^a^ ± 0.40	7.6^a^ ± 0.38	7.5^a^ ± 0.40
**CM**	6.7^c^ ± 0.26	7.4^a^ ± 0.18	8.0^a^ ± 0.18	7.6^a,b^ ± 0.35	7.3^a^ ± 0.26	7.5^a^ ± 0.38	7.6^a^ ± 0.38	7.2a,b ± 0.37	7.3^b^ ± 0.26	7.5^a^ ± 0.46	8.0^a^ ± 0.18	7.7^a^ ± 0.27	7.6^a^ ± 0.32	7.2^a^ ± 0.27
**5% OB**	7.3^b^ ± 0.26	7.2^a^ ± 0.27	7.8^a^ ± 0.23	7.2^b^ ± 0.27	6.8^b^ ± 0.27	6.8^b^ ± 0.27	8.1^b^ ± 0-24	7.1^a,b^ ± 0.24	6.3^d^ ± 0.27	7.4^a^ ± 0.18	7.8^a^ ± 0.27	7.7^a^ ± 0.27	7.0^b^ ± 0.18	7.2^a^ ± 0.27
**10% OB**	6.7^c^ ± 0.27	6.8^b^ ± 0.26	7.2^b^ ± 0.27	7.0^c^ ± 0.18	5.8^c^ ± 0.27	5.8^d^ ± 0.24	6.8^c^ ± 0.27	7.8^a^ ± 0.27	6.7^d^ ± 0.27	7.0^a,b^ ± 0.18	6.7^b^ ± 0.27	7.0^b,c^ ± 0.18	7.0^b^ ± 0.18	6.1^b^ ± 0.24
**15% OB**	6.8^b,c^ ± 0.27	5.8^c^ ± 0.26	7.1^b^ ± 0.35	7.0^c^ ± 0.18	5.7^c^ ± 0.27	5.8^d^ ± 0.26	6.6^c^ ± 0.27	6.7^b,c^ ± 0.27	6.1^e^ ± 0.23	6.6^b^ ± 0.23	6.4^b^ ± 0.41	7.2^a,c^ ± 0.27	6.8^b^ ± 0.23	5.6^b,c^ ± 0.23
**20% OB**	7.1^b,c^ ± 0.26	6.0^c^ ± 0.18	7.0^b^ ± 0.18	7.0^c^ ± 0.18	5.2^d^ ± 0.27	4.6^c^ ± 0.38	5.0^d^ ± 0.24	6.5^c^ ± 0.40	4.6^c^ ± 0.34	6.5^b^ ± 0.40	7.0^b^ ± 0.19	6.5^b^ ± 0.28	6.6^b^ ± 0.38	5.4^c^ ± 0.34

^a–d^ Mean in the same column followed by different superscript letters differ significantly (p < 0.05).

### 3.2. Step 2—Effect of Hydrocolloids on Quality of Maize Spaghetti Enriched with 20% of Oat Bran

#### 3.2.1. Rheological Measurement

In the second experimental step, the spaghetti sample with 20% oat bran was chosen as a reference; in fact, the sensorial parameters of this sample were at the limit of acceptability. In this step, hydrocolloids such us carboxymethylcellulose (CMC), guar seed flour (GUAR), hydroxypropyl cellulose (HPC), gum xanthan (XAN), tapioca starch (TAP), chitosan (CHIT), Agar (AG) and white egg (ALB) were added in order to improve the quality of the selected sample.

The rheological behavior of the dough depends on several factors such as the nature of the protein matrix and its amount, the interaction between the protein matrix and the additives, starch, fat and fibre content, process conditions. Water plays an important role in determining the viscoelastic properties of dough due to its influence on the development of the gluten protein network [36]. In the case of doughs based on gluten-free flour, dairy proteins and hydrocolloids can be used to mimic the viscoelastic properties of gluten and result in improved structural feeling in the mouth, acceptability and shelf life [15]. Based on the chemical structure, they can interact and form complexes with starch, protein, shortening and water.

**Figure 2:**
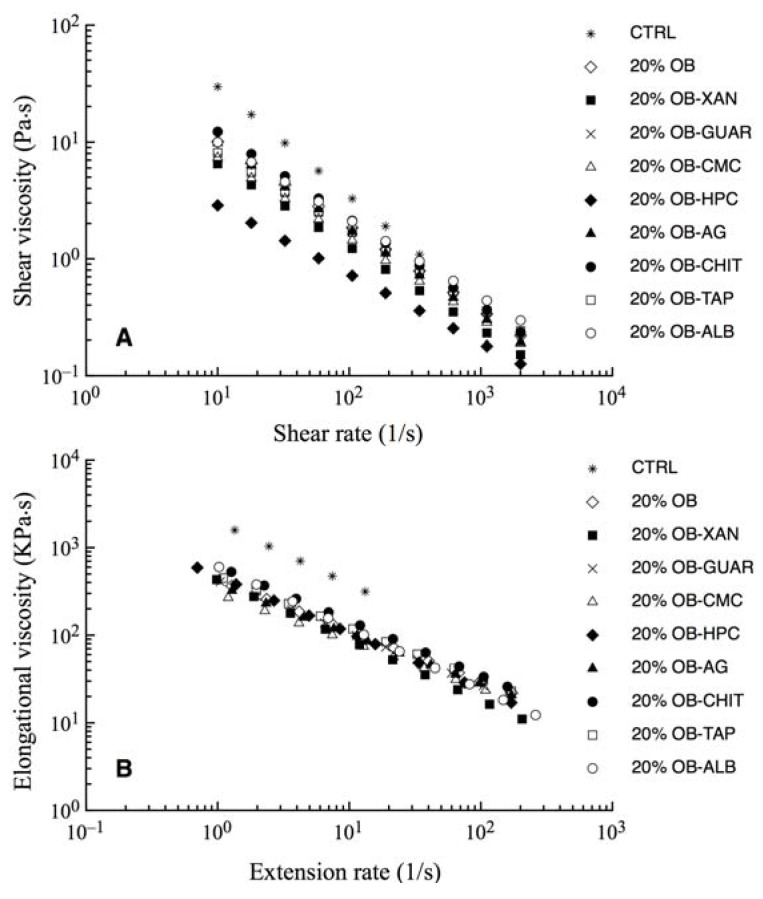
Rheological behavior of the dough samples added with different kinds of hydrocolloids. (**A**) Shear viscosity as a function of shear rate; (**B**) Elongation viscosity as a function of extension rate.

As discussed above, a 20% OB sample is not able to form a solid starch structure; in fact, the presence of oat bran at high concentration would not be able to form a structure only with starch [13]. In the presence of polymers like gum and protein the attractive forces increase [37]. All gums, or hydrocolloids, have a thickening or viscosity producing effect when dispersed in a water medium which gives them an ability to act as a body forming, stabilizing and emulsifying agent [38]. The addition of gum and proteins to the dough system, allows to the starch granules to adhere to one another, moreover water is more homogeneously distributed through the dough system [13]. The gums addition could help the starch released during the process of gelatinization to form a solid physical network. Figure 2 shows the shear and elongation viscosity plotted as a function of shear rate and extension rate, respectively, for dough samples produced in the second step. Also in this case, the beforehand mentioned viscosities decline with the extension and shear rate, denoting a pseudoplastic behavior.

As can be inferred from data shown in Figure 2 all the enriched samples had similar values of elongation and shear viscosity. Similarly, the consistency indices L and K of 20% OB sample (Table 4), obtained by fitting Equations 1 and 2 to the experimental data, showed values similar to those of samples loaded with structuring agents. In particular, the samples added with chitosan and white egg showed the higher value of the L index with respect to the 20% OB sample; whereas the sample added with Agar had the higher value of K index with respect to the sample 20% OB. On the other hand, the CTRL sample showed the highest values of both elongation and shear viscosity with respect to the other samples, in fact Table 4 highlighted that this sample had the highest values of the consistency indices L and K.

**Table 4:** Values of the consistency indices (L and K) and the flow indices (m and n) obtained by fitting Equations 1 and 2 to the experimental data.

	*L*	*m*	*K*	*n*
CTRL	1957.52^a^	0.2921^a^	253.87^a^	6.56E-02^a^
20% OB	420.90^b^	0.4260^b,e^	53.09^b,c^	0.278^b,c^
20% OB-XAN	426.65^b^	0.3142^c^	33.41^b^	0.2904^b,c^
20% OB-GUAR	419.15^b^	0.406^b,d^	34.82^b,c^	0.3236^b^
20% OB-CMC	295.81^c^	0.46^e^	36.22^b,c^	0.3085^b,c^
20% OB-HPC	469.06^d^	0.3545^c^	11.11^e^	0.4111^d^
20% OB-AG	370.95^e^	0.4386^b,e^	54.16^c,d^	0.2590^c^
20% OB-CHIT	613.58^f^	0.3757^c,d^	68.86^d^	0.2521^c^
20% OB-TAP	475.73^d^	0.4106^b,d^	37.40^b,c^	0.3358^b^
20% OB-ALB	609.52^f^	0.2982^a^	46.15^b,c^	0.3356^b^

^a–f^ Mean in the same column followed by different superscript letters differ significantly (p < 0.05).

Results of the rheological analysis obtained in this experimental step, showed a good aptitude of all samples to be processed to produce pasta due to a good mechanical energy value required to extrude and a good rate of dough extrusion; moreover, the addition of hydrocolloids and egg white to the dough enriched with 20% oat bran did not cause any substantial difference in the viscoelastic properties, and in consistency, with respect to the sample without thickening. The differences in the rheological behavior of the investigated hydrocolloids and egg white most probably can be due to changes in granule gelatinization, to gum solubilization, or to starch-gum interactions [39]. In particular, the starch-gum interactions can be affected by the presence of the gum charges and by the affinity between certain gums and the examined starch [39].

#### 3.2.2. Sensory Analysis

The sensorial properties as determined by trained panelists are listed in Table 5 and 6 for fresh-extruded, and dry spaghetti respectively. Results highlighted that the addition of hydrocolloids and white egg improved the sensorial quality of spaghetti in most of the cases. In fact, the sensorial data showed that the uncooked fresh-extruded spaghetti samples with hydrocolloids had an overall quality values higher than that of 20% OB sample, whereas the samples added with XAN and GUAR are statistically similar to the 20% OB sample (Table 5).

The presence of hydrocolloids also improved the sensorial properties of cooked spaghetti samples. In fact, the overall quality value of the fresh cooked samples loaded with hydrocolloids were higher than that of 20% OB sample; whereas, the spaghetti samples with TAP and GUAR showed a lower overall quality. In particular, the sample with TAP had a score under the acceptability threshold for elasticity, bulkiness and adhesiveness. This result can be due to the fact that TAP did not form a stable network structure that entraps starch granules and so it is released during cooking. On the other hand, the fresh-extruted cooked sample with CHIT had the highest overall quality with respect to the samples with other structuring agents due to the high values of elasticity, bulkiness and adhesiveness. Most probably, this is due to the good affinity between CHIT and pregelatinized starch that form a continuous network improving the overall quality.

The sensorial properties of the dry, cooked and uncooked spaghetti samples are listed in Table 6. With regard to dry uncooked spaghetti, samples added with CMC and CHIT showed the highest overall quality value among the samples tested in the second step. Regarding the dry uncooked spaghetti loaded with CMC, color, homogeneity and resistance to break seem to be the main responsible for the increase in the sample overall quality. This result can be due to the fact that CMC had a synergistic effect when blended with non-ionic polymer by providing a considerable increase in viscosity [40]. Shi and BeMiller [39] reported that CMC, such as CHIT, had stronger affinity for starch and form a continuous network, resulting in a greater final viscosity. The dry uncooked sample loaded with TAP had the highest overall quality score compared to the uncooked fresh pasta (*i.e.*, the overall quality score increased from 4.5 to 6.7). This behavior can be ascribed to the different dependence of the hydrocolloids from the network mechanical properties of uncooked dry and fresh spaghetti. In fact, the quality of uncooked dry spaghetti is influenced from mechanical properties of dry network (*i.e.*, break resistance); while the quality of uncooked fresh spaghetti is influenced from the mechanical properties of the hydrated network (*i.e.*, Adhesiveness). Therefore, most probably the thermal process is the factor mainly responsible for the modifications of the pasta structure (Mastromatteo *et al*. 2011) [41].

As can be observed in Table 6, the sensorial parameters of dry cooked spaghetti added with CHIT, CMC, AG and TAP had the higher overall quality value with respect to the 20% OB sample (dry and cooked). In particular, results obtained for spaghetti added with CMC can be due to the fact that the presence of hydrocolloids slows down the diffusion of the amylose molecules from the inner part of spaghetti strand to the surface [14]. Moreover, the positive effect of CHIT on the quality of the dough may be due to the fact that it had high affinity with the pregelatinized starch. In particular, the CHIT and pregelatinized starch form a continuous network that enhances the viscosity of dough.

**Table 5:** Sensory characteristics of fresh-extruded, cooked and non-cooked, spaghetti samples added with 20% oat bran and hydrocolloids.

**Sample**	**Non-cooked spaghetti**				**Cooked spaghetti**									
	Color	Homogeneity	Odor	Overall quality	Elasticity	Firmness	Fibrous	Bulkiness	Adhesiveness	Color	Homogeneity	Odor	Taste	Overall quality
**CTRL Extruded**	7.5^a^ ± 0.17	8.8^a^ ± 0.27	8.1^a^ ± 0.24	8.0^a^ ± 0.17	6.6^a^ ± 0.24	6.7^a,b^ ± 0.26	8.0^a^ ± 0.17	6.7^a^ ± 0.26	6.2^a^ ± 0.26	7.0^a,b^ ± 0.17	8.0^a^ ± 0.17	7.2^a,b,c^ ± 0.26	7.0^a^ ± 0.17	7.1^a^ ± 0.24
**20% OB** **Extruded**	7.0^b^ ± 0.18	6.4^b^ ± 0.23	7.0^b^ ± 0.37	6.5^c^ ± 0.18	5.2^d^ ± 0.27	5.0^c^ ± 0.18	4.4^b^ ± 0.23	7.0^a,b^ ± 0.18	6.1^a^ ± 0.38	6.5^b^ ± 0.38	7.0^b,c^ ± 0.18	6.8^a,b,d^ ± 0.23	6.8^a^ ± 0.23	5.4^b^ ± 0.18
**20% OB-XAN** **Extruded**	7.0^b^ ± 0.18	6.4^b^ ± 0.23	7.0^b^ ± 0.38	6.5^c^ ± 0.18	6.5^a,b^ ± 0.18	6.2^d,e^ ± 0.27	5.2^c^ ± 0.27	7.0^a,b^ ± 0.18	6.5^a,b^ ± 0.18	7.1^a^ ± 0.24	6.4^b^ ± 0.45	7.0^a,b,c,d^ ± 0.18	7.0^a^ ± 0.18	6.4^e,f^ ± 0.18
**20%OB-GUAR** **Extruded**	7.1^a,b^ ± 0.23	6.4^b^ ± 0.23	7.0^b^ ± 0.38	6.1^d^ ± 0.23	5.0^c^ ± 0.18	5.0^c^ ± 0.18	5.0^c^ ± 0.18	7.0^a,b^ ± 0.18	6.3^a,b^ ± 0.27	6.6^b^ ± 0.24	6.4^b^ ± 0.45	6.6^d^ ± 0.24	6.5^d^ ± 0.18	5.1^b^ ± 0.24
**20% OB-CMC** **Extruded**	7.1^a,b^ ± 0.23	7.0^c^ ± 0.18	7.7^a^ ± 0.45	7.5^e^ ± 0.18	6.5^a,b^ ± 0.18	6.2^d,e^ ± 0.26	5.0^c^ ± 0.18	7.2^b^ ± 0.27	6.4^a,b^ ± 0.44	7.0^a,b^ ± 0	7.2^c^ ± 0.26	7.2^a,b,c^ ± 0.26	7.2^a,b^ ± 0.26	6.4^e,f^ ± 0.18
**20% OB-HPC** **Extruded**	7.5^a^ ± 0.18	7.2^c^ ± 0.26	7.1^b^ ± 0.44	7.2^e^ ± 0.27	6.4^a,b^ ± 0.23	6.0^e^ ± 0.18	5.0^c^ ± 0.18	6.8^a^ ± 0.23	6.4^a,b^ ± 0.23	6.7^a,b^ ± 0.27	7.1^c^ ± 0.23	6.6^d^ ± 0.23	7.0^a^ ± 0.18	6.4^e^ ± 0.18
**20% OB-AG** **Extruded**	7.5^a^ ± 0.18	7.2^c^ ± 0.26	7.1^b^ ± 0.44	7.5^e^ ± 0.18	6.9^a^ ± 0.23	6.4^b,d^ ± 0.23	5.0^c^ ± 0.18	7.2^b,c^ ± 0.26	6.7^b^ ± 0.27	6.7^a,b^ ± 0.27	6.8^b,c^ ± 0.23	6.7^b,d^ ± 0.27	7.0^a^ ± 0.18	6.7^e^ ± 0.27
**20% OB-TAP** **Extruded**	7.2^a,b^ ± 0.26	7.2^c^ ± 0.26	8.0^a^ ± 0.18	7.2^e^ ± 0.27	4.5^c^ ± 0.18	4.2^f^ ± 0.27	5.0^c^ ± 0.18	4.0^d^ ± 0.18	4.0^c^ ± 0.18	6.7^a,b^ ± 0.27	6.8^b,c^ ± 0.23	7.2^a,c^ ± 0.26	7.0^a^ ± 0.18	4.5^d^ ± 0.18
**20% OB-ALB** **Extruded**	7.0^a,b^ ± 0.18	7.2^c^ ± 0.27	8.0^a^ ± 0.18	7.4^e^ ± 0.18	6.2^b^ ± 0.26	6.0^e^ ± 0.18	5.0^c^ ± 0.18	6.2^e^ ± 0.26	5.2^d^ ± 0.26	7.0^a,b^ ± 0.18	7.2^c^ ± 0.27	7.4^c^ ± 0.23	6.8^a^ ± 0.23	6.1^f^ ± 0.23
**20% OB-CHIT** **Extruded**	6.8^b^ ± 0.23	7.2^c^ ± 0.27	7.8^a^ ± 0.26	7.4^e^ ± 0.18	6.8^a^ ± 0.26	7.0^a^ ± 0.18	5.0^c^ ± 0.18	7.6^c^ ± 0.18	7.4^e^ ± 0.18	6.8^a,b^ ± 0.23	7.2^c^ ± 0.27	7.4^c^ ± 0.23	7.4^b^ ± 0.23	7.4^a^ ± 0.23

^a–f^ Mean in the same column followed by different superscript letters differ significantly (p < 0.05).

**Table 6:** Sensory characteristics of dry cooked and non-cooked spaghetti samples added with 20% oat bran and hydrocolloids.

**Sample**	**Non-cooked spaghetti**				**Cooked spaghetti**									
	Color	Homogeneity	Resistance to break	Overall quality	Elasticity	Firmness	Fibrous	Bulkiness	Adhesiveness	Color	Homogeneity	Odor	Taste	Overall quality
**CTRL**	8.0^a^ ± 0.18	7.4^a^ ± 0.18	8.0^a^ ± 0.18	8.0^a^ ± 0.18	7.3^a,b^ ± 0.38	7.7^a^ ± 0.40	8.0^a^ ± 0.18	7.5^a,b^ ± 0.40	8.0^a,f^ ± 0.18	7.5^a^ ± 0.40	8.0^a^ ± 0.18	7.7^a^ ± 0.40	7.6^a^ ± 0.38	7.5^a,e^ ± 0.40
**CM**	6.8^d^ ± 0.26	7.4^a^ ± 0.18	8.0^a^ ± 0.18	7.6^a,b^ ± 0.35	7.3^a,b^ ± 0.26	7.5^a,b^ ± 0.38	7.5^a^ ± 0.18	7.2^b,c^ ± 0.37	7.3^b^ ± 0.26	7.5^a^ ± 0.46	8.0^a^ ± 0.18	7.7^a^ ± 0.27	7.6^a^ ± 0.32	7.3^a,b^ ± 0.26
**20% OB**	7.1^b,c,d^ ± 0.26	6.0^c^ ± 0.18	7.0^c^ ± 0.18	7.0^c,d^ ± 0.18	5.2^c^ ± 0.27	4.6^d^ ± 0.38	5.0^b^ ± 0.24	6.5^d^ ± 0.40	4.6^c^ ± 0.34	6.5^c^ ± 0.40	7.0^b^ ± 0.19	6.5^b^ ± 0.28	6.6^c^ ± 0.38	5.4^d^ ± 0.34
**20%OB-CMC**	7.0^b,c,d^ ± 0.18	7.1^a,b^ ± 0.18	7.8^a^ ± 0.23	7.8^a^ ± 0.23	7.0^b,f^ ± 0.40	7.1^b,c^ ± 0.24	5.0^b^ ± 0.18	8.0^a^ ± 0.18	7.7^a,b^ ± 0.27	7.1^a,b^ ± 0.27	7.2^b^ ± 0.27	7.2^a^ ± 0.40	7.0^c^ ± 0.18	7.2^a,b^ ± 0.27
**20% OB-CHIT**	7.4^c^ ± 0.23	7.0^a,b^ ± 0.18	7.4^b^ ± 0.18	7.4^b^ ± 0.23	7.8^a,g^ ± 0.26	7.8^a^ ± 0.18	5.0^b^ ± 0.18	7.8^a,b^ ± 0.27	7.8^a,b^ ± 0.26	7.1^a,b^ ± 0.24	7.2^b^ ± 0.27	7.4^a^ ± 0.45	7.0^c^ ± 0.18	7.8^e^ ± 0.27
**20% OB-ALB**	7.0^b,c,d^ ± 0.18	6.8^b^ ± 0.23	6.8^d,c^ ± 0.23	6.8^e^ ± 0.23	5.3^c^ ± 0.24	5.8^e^ ± 0.24	5.0^b^ ± 0.18	5.2^e^ ± 0.27	4.2^c^ ± 0.27	6.6^b,c^ ± 0.24	7.2^b^ ± 0.27	6.1^b^ ± 0.24	5.8^b^ ± 0.38	5.3^c^ ± 0.27
**20% OB-XAN**	6.9^c,d^ ± 0.23	6.8^b^ ± 0.23	7.4^b^ ± 0.23	7.3^b^ ± 0.26	6.4^d,e^ ± 0.24	6.6^c^ ± 0.24	5.0^b^ ± 0.18	7.3^b,c^ ± 0.27	7.2^g,f^ ± 0.27	7.2^a,b^ ± 0.26	7.2^b^ ± 0.27	7.2^b^ ± 0.27	7.0^c^ ± 0.18	6.8^b,d^ ± 027
**20% OB-HPC**	6.9^c,d^ ± 0.23	6.8^c^ ± 0.23	6.4^e^ ± 0.23	6.4^d^ ± 0.23	6.0^d^ ± 0.18	5.0^d^ ± 0.18	5.0^b^ ± 0.18	6.8^c,d^ ± 0.24	6.2^d^ ± 0.27	7.2^a,b^ ± 0.26	7.2^b^ ± 0.27	7.2^b^ ± 0.27	7.0^c^ ± 0.18	5.7^c^ ± 0.27
**20% OB-TAP**	7.4^b^ ± 0.23	7.2^a,c^ ± 0.27	7.4^b^ ± 0.23	6.7^c,d^ ± 0.23	7.6^a^ ± 0.24	7.6^a,b^ ± 0.24	5.0^b^ ± 0.18	7.4^a,b^ ± 0.18	6.8^f,e^ ± 0.27	7.2^a,b^ ± 0.26	7.2^b^ ± 0.27	7.2^b^ ± 0.27	7.0^c^ ± 0.18	7.1^a,b^ ± 0.24
**20% OB-AG**	7.2^b,c,d^ ± 0.27	7.1^a,b^ ± 0.23	6.5^d,e^ ± 0.38	6.8^c,d^ ± 0.27	7.5^a,b^ ± 0.28	7.5^a,b^ ± 0.28	5.0^b^ ± 0.18	7.3^b,c^ ± 0.26	6.7^e^ ± 0.27	7.2^a,b^ ± 0.26	7.2^b^ ± 0.27	7.2^b^ ± 0.27	7.0^c^ ± 0.18	7.0^b^ ± 0.18
**20% OB-GUAR**	7.3^b,c^ ± 0.26	7.1^a,b^ ± 0.26	6.7^c,d,e^ ± 0.26	6.8^c,d^ ± 0.26	6.6^e,f^ ± 0.24	6.6^c^ ± 0.24	5.0^b^ ± 0.18	7.4^a,b^ ± 0.18	6.2^d^ ± 0.27	7.2^a,b^ ± 0.26	7.2^b^ ± 0.27	7.2^b^ ± 0.27	7.0^c^ ± 0.18	6.3^d^ ± 0.24

^a–d^ Mean in the same column followed by different superscript letters differ significantly (p < 0.05).

The sensorial data showed that the spaghetti added with egg white (ALB) had a lower overall quality. This result could be due to the fact that the absence of pregelatinized starch did not allow the formation of a continuous network with ALB. In conclusion, the spaghetti overall quality was high when in the dough formulation there were hydrocolloids with charge. This behavior could be due to the fact that hydrocolloids in presence of charge have more affinity with the pregelatinized starch and so form a stable polymeric structure that improves the rheological and sensorial properties of pasta.

This result could be due to the fact that hydrocolloids in presence of charge can form a stable polymeric structure that improves the rheological and sensorial properties of pasta.

## 4. Conclusions

The optimization of the formulation of gluten-free functional spaghetti based on maize flour and oat bran enriched in β-glucans (22%) was addressed in this study. In the first experimental step, the oat bran amount added to spaghetti was continuously increased until the overall sensory quality of oat bran-loaded spaghetti decreased to the sensory threshold value, which was reached at an oat bran concentration of 20%. At the second experimental step, structuring agents were used to improve the overall sensory quality of 20% oat bran-loaded maize spaghetti. The rheological properties of dough and the sensorial attributes of the spaghetti samples were evaluated. In particular, results of the rheological analysis obtained in this experimental step, showed a good aptitude of all samples to be processed to produce pasta. Moreover, the addition of hydrocolloids and egg white to the dough enriched with 20% oat bran did not cause any substantial difference in the viscoelastic properties, consistently, with respect to the sample, without thickening. Most of the structuring agents used in this work improved the sensory characteristics of the spaghetti samples, which showed good elasticity and firmness, low adhesiveness and bulkiness. Moreover, the structuring agents did not alter odor and taste of the samples that was pleasant despite the high percentage of oat bran. In particular, the best results were obtained on both fresh and dry spaghetti by means of the addition of carboxymethylcellulose and chitosan to the dough formulation. In conclusion, the pregelatinization of maize flour combined with the use of structuring agents, could be a useful tool to obtain oat-enriched gluten-free pasta with sensory properties close to the unloaded maize sample.
